# Interception Systems in Assessment of Dermal Exposure to Pesticides: Laboratory Comparison of Media

**DOI:** 10.3390/ijerph17124389

**Published:** 2020-06-18

**Authors:** Elena Barrese, Giovanna Tranfo, Antonella Marramao, Marialuisa Scarpelli

**Affiliations:** 1 INAIL, Department of Occupational and Environmental Medicine, Epidemiology and Hygiene (DiMEILA), Area Industriale Papa Benedetto XVI, 88046 Lamezia Terme (CZ), Italy; e.barrese@inail.it (E.B.); a.marramao@inail.it (A.M.); m.scarpelli@inail.it (M.S.); 2 INAIL, Department of Occupational and Environmental Medicine, Epidemiology and Hygiene (DiMEILA) Via Di Fontana Candida 1, 00078 Monte Porzio Catone (RM), Italy

**Keywords:** agrochemicals, wipe sampling, sampling pads, occupational exposure, cute

## Abstract

Dermal exposure of workers to pesticides can be assessed using patches, placed on the workers’ clothes or used to wipe off the substance from the skin. Since there are no official indications of the materials to be used for patch sampling, a wide range of materials is suggested in the scientific literature. This paper reports a laboratory study on the affinity of four different pesticides widely used in southern Italy with five patch matrices. Imidacloprid, Hexythiazox, Boscalid and Myclobutanil were tested with cotton and gauze sheets, polyethylene tissue and two different grades of cellulose papers. An aerosol machine was used to nebulize the substance on the patches in a closed system, simulating the conditions of use on the workers’ clothes. The recovery of the analytes from the media was evaluated, by spiking the patches with a known amount of each active substance and testing their performances as skin wipes. Samples were extracted and analyzed in gas chromatography with an electron capture detector. The recovery from the spiked patches was 89–96% for all pesticides, while the test recoveries were very different. Results showed a higher affinity with Imidacloprid and Hexythiazox for gauze, with Myclobutanil for cotton and with Boscalid for paper filters (W41).

## 1. Introduction

Pesticides are widely used in modern agriculture to increase the yields of production. Because of their high biological activity, and, in some cases, long persistence in the environment, pesticides may cause harmful effects to human health and to the environment [1]. Occupational exposure typically occurs in workers involved in the manufacture of pesticides and in the agricultural sector, among farmers and professional applicators of pesticides [2]. In recent years, the assessment of occupational exposure to chemicals via the dermal route has grown in importance, in particular for agricultural workers. The current understanding of dermal exposure and uptake has come from researchers investigating the health effects of pesticides [3].

Contact with pesticides can occur the during mixing and loading of products, the application of the spray mixtures and the cleaning of the equipment. Dermal absorption is an important route of exposure in these activities, especially in the mixing and loading, because here the concentrated products are used. Dermal exposure is also significant in closed systems, such as the agricultural greenhouse, where dangerous accumulations of contaminants could occur. The degree of hazard depends on the toxicity of the pesticide, time of exposure and pesticide formulation: powders, dusts and granular pesticides are not absorbed as easily through the skin as liquid formulations, and formulations containing organic solvents and oil-based pesticides are usually absorbed more quickly. Further, the body part contaminated has its role, as certain body areas are more prone to absorption than other areas [4].

Exposure depends also on how the spillage occurs, and on the spraying equipment used. Hand spraying with wide-area spray nozzles is associated with greater exposure for the operators than narrowly focused spray nozzles. Pesticide deposition on different parts of the operator’s body depends also on the individual work habits; the hands and the forearms experience the greatest contamination during the preparation and application of pesticides. Re-entry farm workers may be more significantly exposed than applicators, because of lower risk perception, less training on the use of personal protective equipment (PPE), and greater duration of exposure [2]. Occupational exposure becomes significant in closed spaces such as greenhouses, where the high temperature and relative humidity can increase skin absorption [5].

Because of the many possible variables that can influence dermal absorption and the efficiency of sampling methods, the quantitative assessment of dermal exposure is a complex and challenging task [6]. International organizations [7,8] and the scientific literature [9,10,11] indicate different approaches to the quantitative assessment of this type of exposure.

Direct methods include surrogate skin and patch methods, using whole body suits or representative patches to capture the potential exposure, and fluorescence visualization of materials deposited or retained on the skin surface. Washing, wiping or skin stripping techniques can be used to indirectly determine the amount of material present on the skin at a given time point, and biomonitoring to provide an estimate of the amount of actual internal exposure.

Both patch methods and skin wiping involve the use of materials that selectively capture the considered substance and then release it to be analyzed appropriately. The assessment of potential dermal exposure is performed by applying the patches over the clothes, while the effective one is estimated by applying the patches directly onto the skin, under the clothes of the operators, or removing the active substances deposited on the skin. The quantity of a pesticide on a patch with a known area is related to the area of the limb or other body part using standard body part surface areas. Individual body part exposure values are then summed up to give a total potential dermal exposure, that can be expressed in mg/h, mg/d or mg/kg of product handled or applied.

Patches usually consist of two layers, the sampling one and a second one made of an impervious material, such as aluminium or plastic foil, to reduce the potential contamination of the skin, or a loss of pesticide through the patch. The typical thickness of the patches is approximately 1 mm, and the most commonly used size is 10 × 10 cm^2^. They are applied to the worker’s body immediately before they start working, and are removed after the end of the working activity. A wide range of materials are used for the scope. Durham and Wolfe [12] published the first review paper detailing methods for measuring dermal exposure to pesticides. Some authors and institutions recommend α-cellulose paper, whereas others suggest alternative materials [13,14], such as cotton, or both polyester and cotton [15]. Filter paper was used for Imidacloprid dermal exposure evaluation [16]. The UK Health and Safety Executive (HSE) [17] recommends using several different patch materials (fabric, polymer, paper, charcoal cloth or composite materials) instead of one material for all substances. It was shown that different substances could have different recovery efficiencies with the same material [18]. Temperature, humidity and the chemical-physical characteristics of substances are variables that must be considered, because they could influence the sampling performances.

The aim of this work was to evaluate the most suitable matrices for performing potential dermal exposure evaluation (PDE), during the application of four chlorinated pesticides widely used in the countryside area of Lamezia Terme. Imidacloprid, Hexythiazox, Boscalid and Myclobutanil are active substances (a.s.) intensively used in the working environments typical of southern Italy, and many agricultural workers are exposed to them on a daily basis. Normally, an aqueous mixture is prepared, to be used with the manual lance or with air-blaster sprayers, depending on the kind of crop or on the workplace area. This paper describes a laboratory study that evaluates the affinity of five of the most-used patch matrices (cotton and gauze sheets, polyethylene tissue and two different grades of cellulose paper) with the four above mentioned organochlorine pesticides.

Imidacloprid is a chlorinated compound, structurally classified as a chloronicotinyl nitroguanidine [19], a novel class of pesticides chemically related to nicotine, which binds to insect cholinergic receptors, causing death at sufficient concentrations [20], and is selectively toxic to arthropods and relatively non-toxic to vertebrates. Under WHO [21] classification, Imidacloprid is in Class II; moderately hazardous. U.S. Environmental Protection Agency (EPA) [22] has classified Imidacloprid into Group E, ‘evidence of non-carcinogenicity for humans’. The European Food Safety Authority (EFSA) [23] has delivered its scientific opinion at the request of the European Commission on Imidacloprid, concluding that some levels of acceptable exposure to Imidacloprid might not be protective enough against developmental neurotoxicity. Some cases of poisoning have been also described in the literature [24]. In 2018, the European Commission banned the outdoor use of Imidacloprid because of potential damage to bees, and its use is only allowed in greenhouses [25].

Hexythiazox is an acaricide that acts against the egg, larval and nymph stages. It is classified in group C of U.S. EPA Cancer Classification: ‘possible human carcinogen’ [26]. The human toxicity excerpts are irritation of eyes, nose, throat and skin.

Boscalid belongs to the group of aromatic heteromonocyclic compounds, a fungicide active against a broad range of fungal pathogens. EPA [27] classified Boscalid with “suggestive evidence of carcinogenicity, but not sufficient to assess human carcinogenic potential”, and therefore the quantification of human cancer risk is not mentioned.

Myclobutanil is a triazole chemical used as a fungicide. It is stable under recommended storage and normal use conditions, but can decompose at elevated temperatures. Generation of gas during decomposition can cause pressure build-ups in closed systems. Decomposition products depend on temperature, air supply and the presence of other materials, but can include carbon monoxide, carbon dioxide, hydrogen chloride, hydrogen cyanide and nitrogen oxides [28]. The primary target organ following exposure to Myclobutanil is the liver. Skin irritation and/or gross and microscopic changes of the treated skin were observed after application of Myclobutanil formulations at 100 mg/kg bw/day. The NOAEL (no observed adverse effect level) for local effects is 10 mg/kg bw/day, whereas for systemic toxic effects it is 100 mg/kg bw/day [29].

The compounds studied have also been associated with health problems due to cutaneous exposure [30]. Given their very low volatility, the amount of material inhaled is likely to be scarce, while the dermal exposure can be significant. In assessing dermal exposure, sampling is the first and the most important step, followed by sample extraction and analysis.

## 2. Materials and Methods

All reagents were of analytical grade. The active substances of the selected pesticides were PESTANAL grade, purchased from Sigma Aldrich (Milan, Italy). All solvents used for sample processing and analysis were of GC grade. SPE cartridges Supelclean TM columns LC18 (500 mg, 6 mL) were purchased from Sigma Aldrich (Milan, Italy). Calibration standards have been prepared by serial dilution from stock solutions in acetone. The mixtures used for the real use were water solutions in the case of Hexythiazox and Imidacloprid, and aqueous suspensions in the case of Boscalid and Myclobutanil, as a concentration higher than the water solubility of the active substances was used. The pure standards were dissolved or suspended in water in order to reproduce these mixtures (Mixture concentrations are reported in Table 1, together with other properties of the active substances). Temperature and humidity were monitored with a portable hygrometer (Thermo Hygrometer RS1360A, RS PRO Sesto San Giovanni MI, Italy).

### 2.1. Patches Preparation

**Figure 1:**
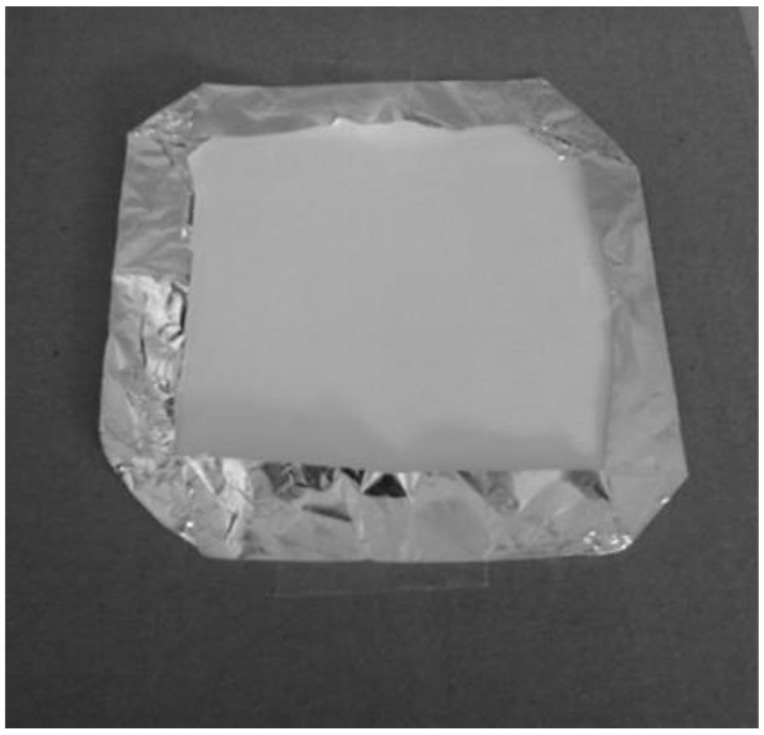
Example of a paper patch with the aluminium foil backing.

The patches were made by cutting large sheets into 10.2 cm × 10.2 cm squares, which were used alone for the wiping recovery test, and fixed to a protective backing of aluminium foil, as suggested by the U.S. EPA Residential Exposure Test Guidelines [31] for the aerosol exposure test (Figure 1).

The five media consisted of:

1. cellulose filter paper sheets grade 41 (Whatman™ Article No. 28418027), pore size 20–25 μm (Particle retention)
2. cellulose filter paper standard grade 1 (Whatman™ Article No. 28413923), pore size 11 μm (Particle retention)
3. cotton wipes (TexWipe^®^ Cotton TX304)
4. layers of surgical gauze patches (Dynarex)
5. polyethylene tissue, a synthetic material used to produce protective overalls (Tyvex^®^ Du Pont)

A single sheet of white filter paper was used for each cellulose patch; the same was done for cotton and polyethylene sheets, while for the gauze, three sheets of the same size were overlapped to reach the desired thickness (1 mm).

### 2.2. Analytical Method

The four pesticides were determined by means of a Gas Chromatograph AutoSystem XL GC equipped with an Electron Capture Detector (ECD/GC) (Perkin Elmer Instruments, Waltham, Massachusetts, USA). The capillary column was a Perkin-Elmer 1701, 30 m × 0.32 mm × 0.25 μm. Carrier gas was Helium, at a flow rate of 1.5 mL/min. Split injection mode and a temperature gradient were used, from 150 °C to 240 °C, 2 °C/min, and then to 280 °C, 10 °C/min.

The validation of the analytical method was carried out in accordance with the standard of the Italian body for unification of chemical methods, UNICHIM 179/1 “Guidelines for the validation of analytical methods in chemical laboratories”, in accordance with the technical standard UNI CEI EN ISO / IEC 17025: 2005 (General requirements for the competence of testing and calibration laboratories). Single calibration solutions were prepared at four concentration points, ranging from 0.2 to 100 µg/mL, by diluting the commercially available solution of the analyte of interest. Perkin Elmer Totalchrom Software was used for the integration of the areas of the chromatographic peaks generated by the analysis of the calibration standards, to generate a calibration curve. The limit of detection (LOD) and the limit of quantification (LOQ) were determined for each substance on the calibration curve according to the formulas LOD = 3.3 (Sy/S) and LOQ = 10 (Sy/S), where Sy is the standard deviation of the y-intercepts of regression lines and S is the slope of the calibration curve.

### 2.3. Experimental

All experiments were performed in the same laboratory, under a chemical hood, far from direct sunlight and from any sources of heat. The monitored temperatures and humidity were in the range 25–27 °C and 50–60% respectively.

The direct recoveries of Imidacloprid, Hexythiazox, Myclobutanil and Boscalid from the different matrices were determined by adding 10 mL of an ultrapure water solution/suspension of each a.s., at the concentration of 50 µg/mL, to each matrix patch, which was left to dry and then sonicated for 10 min with 10 mL of acetone, purified on a SPE column, and analyzed by ECD/GC. Two replicates were performed for each test.

The standard pesticide solutions/suspensions were prepared in ultrapure water, at concentrations selected to simulate the aqueous mixture of pesticide applied during the following real field applications, according to the product labels: Myclobutanil for the treatment of zucchini, Imidacloprid for flowers in pot, Boscalid for strawberries and Hexythiazox for the citrus fruit.

Two field simulation experiments were conducted for each a.s. A total of 10 filters were used for each experiment, 2 replicates for each one of the 5 different matrices, for a total of 80 filters (4 replicates for each material/active substance pair).

**Figure 2:**
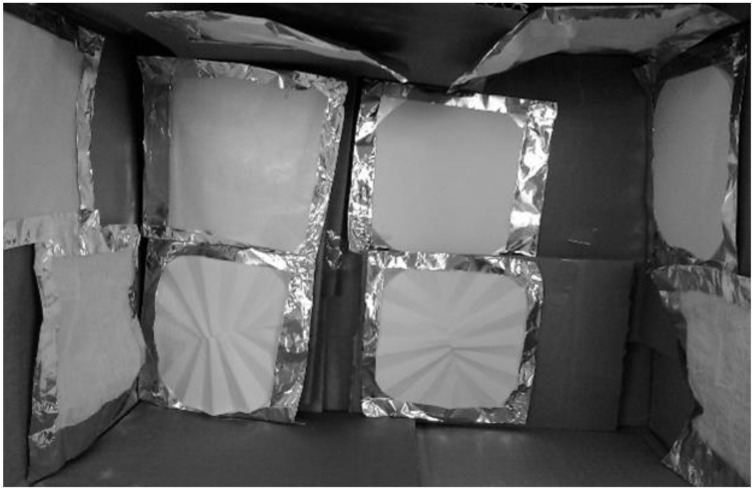
Patches placed inside the test chamber.

In each experiment, 10 patches were stuck to the inner walls of a test chamber. The chamber was a sturdy cardboard box of 30,000 cm^3^ volume (25 cm × 40 cm × 30 cm). The front face was removable to allow easy removal of contaminated patches and the placement of the new ones, while no patches were placed on the box floor, as this area collects all the solution/suspension falling down after being sprayed into the test chamber (Figure 2).

A pneumatic nebulizer for breathing therapy (LAICA S.p.A. Vicenza, Italy) was used to uniformly spray 10 mL of working solution/suspension of one of the four selected pesticide for each experiment through an orifice placed on a face of the box. The air passage through the solution for nebulizing assured the continuous stirring of the suspension, in order to guarantee its homogeneity.

As soon as each solution was consumed (after about 120 min) and the environment was saturated, the machine was turned off and the patches left to dry. Then, the patches were sonicated for 10 min with 10 mL of acetone, and the extracts were cleaned up on a SPE column. The choice of acetone as unique extraction solvent was justified by the common very high solubility of the a.s. in this solvent, as shown in Table 1.

In order to remove the interfering compounds from the pads used in the field, USEPA suggested a variety of cleaning steps (method 8081B). Solid-phase extraction (SPE) is a widely used sample preparation technique. The cartridge was pre-washed with 10 mL of *n*-hexane and subsequently the extracts were eluted [32,33]. The eluates were analysed by ECD/GC.

### 2.4. Statistics

The present investigation does not require a proper statistical analysis, but all calculations and graphic representations have been performed using Microsoft Office Excel Plus 2016. Data are presented as mean, and standard deviations and percentages have been calculated.

## 3. Results

Direct recovery: the mean recovery of the pesticide from the five different patch matrices ranged between 89% and 95.6% for all pesticides, indicating that all of them can be used to wipe off the substances from the skin (Table 2).

Field exposure simulation experiments were conducted as described above, and completed over a few days, keeping the extracted samples in vials at 4 °C. The samples were treated as indicated in the literature for the assessment of dermal exposure [34]. The amount of active substance for each pad was determined. The mean amount of active substance found for the four replicates, with the relative standard deviation, is reported in Table 3.

The recoveries of the active substances can be calculated from the total sprayed amount, assuming a homogeneous distribution of the sprayed mixture inside the chamber, and a normalized recovery can be calculated by assigning the value of 100 to the sum of the recoveries. These values are reported in Table 4.

**Figure 3:**
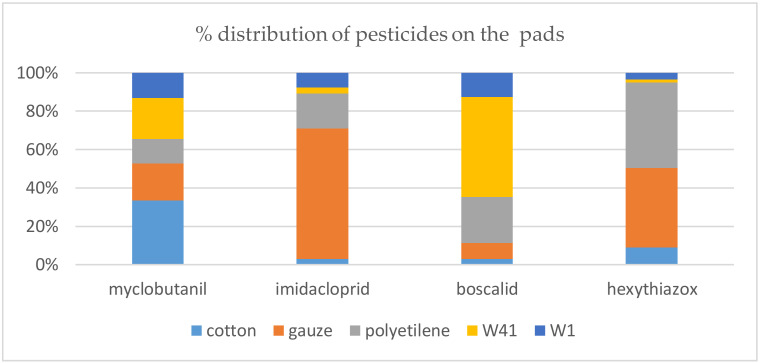
Distribution of each pesticide among the different matrices.

Form the recoveries reported in Table 4, different behaviors are highlighted for each of the five media, and the relative distribution of each pesticide among the different matrices, expressed as the normalized recovery, is visually shown in Figure 3.

The results show that all these materials can be used when skin wiping has to be performed, as they only have to mechanically remove the pesticide from the skin and absorb it by contact. If the materials are used to produce pads, to be placed on the workers clothes to passively absorb the pesticide aerosol, the different affinities of the materials with the active substances play an important role, leading to significantly different results. The gauze seems to be the most appropriate material for Imidacloprid and Hexythiazox, while a material that gives acceptable results for all active substances is polyethylene, which is also used for producing working clothes of farmers. Only Myclobutanil shows an affinity with cotton, and Boscalid with one type of paper filter (W41).

## 4. Discussion

The materials selected to produce the pads are all readily available. Polyethylene fabric is frequently used as a synthetic material for protective suits (Tyvex brand), while cotton and gauze are often used for gloves. Filter papers are commonly used in chemical laboratories. The patches are made in accordance with recommendations by the Organization for Economic Co-operation and Development (OECD) [16]: that the collection area of each patch be approximately 100 cm^2^ in size, as patches smaller than 50 cm^2^ are generally inadequate. The same paper also suggests aluminium foil as a backing material.

Results on direct recovery are in agreement with the findings of Aprea [17], who showed about 100% direct recovery validation of filter paper for Imidacloprid.

The analysis of the materials’ properties could help in understanding their behaviors towards the selected pesticides. Polyethylene consists of nonpolar, saturated, high molecular-weight hydrocarbons, and therefore, it absorbs almost no water, but can absorb some solvent, oil and plasticizers [35]. This can justify its moderate affinity with all the selected active substances that are organic compounds, having low or very low water solubility. On the other hand, the presence of hydroxyl groups on the cellulose macromolecules imparts hydrophilicity to cotton, gauze and filter papers, which preferably absorb the more water-soluble substances. This is verified for the gauze, which preferably retains Hexythiazox and Imidacloprid. Myclobutanil seems to be well retained by cotton, but it must be remembered that this pesticide has a very poor solubility in water, and it is sprayed as an aqueous suspension, so it could be physically retained by the woven-fibers structure more than because of its chemical affinity. The same hypothesis can be made for filter paper W41, which is a cellulose paper for coarse particles, and which showed good recovery for Boscalid.

In accordance with what is found in the literature [36], where gauze is used to make patches for pesticide evaluation, especially for powders and granules, a good affinity with Imidacloprid and Hexythiazox was shown for this material. As Imidacloprid and Hexythiazox are often mixed together in the same solution, the possibility of using the same material as a sampling media is an advantage. When the intent is to use a single matrix that fits all four pesticides for dermal evaluation, surely the gauze or polyethylene seem to have better results, while a specific material can be used if only a single substance is monitored.

Our results are in agreement with J. Davis [37], who confirms that the type of material used for construction of cloth patches may have a considerable effect on the amount of toxicant that they will retain.

There are not so many published laboratory studies to compare with our findings, as field exposure assessment studies take into account other variables, like the different parts of the body where the patch is applied, the comparison with inhalation exposure, and the comparison of the potential exposure with the total applied quantity of pesticide, all for the same active substance [38].

This study has some limitations, as the active substances have been used instead of the formulated products, and even if results give important information, the simulation does not match exactly the field operations. The real airborne concentration of each pesticide during spraying is not measured, but this does not affect the quality of results. In fact, this is a comparison study, and even if all substances and interception media pairs are treated the same way, different behaviors are highlighted. Further investigations will be aimed at determining if the relative affinity of the active substances with the used materials would be influenced by the use of the formulated products in the simulation.

## 5. Conclusions

Despite the great relevance of dermal exposure assessment for agricultural workers, there are not so many papers studying the affinity of different absorbing media with specific active substances, and this is one of the very few papers examining this important issue.

All the tested media can be used when skin wiping has to be performed. If the materials are used to produce pads, significantly different results are found. The gauze is an appropriate material for Imidacloprid and Hexythiazox, polyethylene gives acceptable results for all active substances, Myclobutanil shows an affinity with cotton, and Boscalid with W41 paper filter.

This study provides preliminary indications of the type of materials to be used for the dermal sampling of Imidacloprid, Hexythiazox, Boscalid and Myclobutanil, and suggests a method for the laboratory simulation of field operations, that can be applied to more active substances and to other sampling media.

## Figures and Tables

**Table 1:** Chemical properties of the four pesticides considered.

Active Substance	Molecular Structure	Molecular Mass (g mol^−1^)	Vapour Pressure at 25 °C (mPa)	Acetone Solubility g/L	Water Solubility mg/L	Field Sprayed Concentration mg/L
Hexythiazox	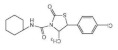	352.88	1.33 × 10^−3^	160	142	64
Imidacloprid	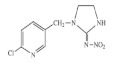	255.66	4.0 × 10^−7^	50	610	86
Boscalid	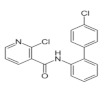	343.21	0.00072	160–200	4.6	214
Myclobutanil	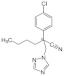	288.78	0.198	50 to 100	0.5	200

**Table 2:** Mean (SD) percent of direct recovery of active substances form the pads (*n* = 2).

Active Substance	Cotton	Gauze	Polyethylene	W41	W1
Myclobutanil	95.1 (0.14)	90.1 (0.07)	89.2 (0.07)	89.7 (0.01)	89.1 (0.11)
Imidacloprid	89.5 (0.21)	95.6 (0.39	90.2 (0.28)	89.5 (0.21)	90.5 (0.07)
Boscalid	89.7 (0.01)	91.1 (0.92)	94.5 (0.11)	90.6 (0.92)	89.2 (0.14)
Hexythiazox	89.3 (0.28)	93.1 (0.07)	92.1 (1.91)	89.1 (0.11)	89 (0.57)

**Table 3:** Mean amount of a.s. (active substance) found on each matrix.

Mean Values in µg/pad (SD)—*n* = 4							
Matrix	Sprayed Amount (µg)	Cotton	Gauze	Polyethylene	W41	W1
Myclobutanil	640	26.83 (2.59)	15.48 (3.31)	10.26 (0.89)	17.15 (7.90)	10.55 (9.09)
Imidacloprid	860	5.41 (0.97)	130.28 (2.80)	34.8 (0.98)	6.0 (0.03)	14.82 (4.38)
Boscalid	2140	39.45 (7.0)	106.68 (5.66)	306.6 (15.63)	672 (29.70)	160.5 (23.26)
Hexythiazox	2000	43 (7.07)	195.92 (15.26)	211.65 (8.56)	6.45 (0.66)	16.82 (2.97)

**Table 4:** Real and normalized recoveries for each patch/substance pair.

Patch Material	Myclobutanil% Recovery	Normalized Recovery	Imidacloprid% Recovery	Normalized Recovery	Boscalid% Recovery	Normalized Recovery	Hexythiazox% Recovery	Normalized Recovery
**Cotton**	4.19	33.41	0.63	2.83	0.28	0.48	2.15	9.08
**Gauze**	2.42	19.30	15.15	68.09	4.99	8.53	9.79	41.34
**Polyethylene**	1.60	12.76	4.05	18.20	14.33	24.50	10.58	44.68
**W41**	2.68	21.37	0.70	3.15	31.40	53.68	0.32	1.35
**W1**	1.65	13.16	1.72	7.73	7.50	12.82	0.84	3.55
**Total**	12.54	100.00	22.25	100.00	58.50	100.00	23.68	100.00
